# Therapeutic Efficacy of Alpha-RIT Using a ^213^Bi-Anti-hCD138 Antibody in a Mouse Model of Ovarian Peritoneal Carcinomatosis

**DOI:** 10.3389/fmed.2015.00088

**Published:** 2015-12-21

**Authors:** Aurélie Derrien, Sébastien Gouard, Catherine Maurel, Marie-Hélène Gaugler, Frank Bruchertseifer, Alfred Morgenstern, Alain Faivre-Chauvet, Jean-Marc Classe, Michel Chérel

**Affiliations:** ^1^Centre Régional de Recherche en Cancérologie Nantes/Angers (CRCNA) UMR892 INSERM, Nantes, France; ^2^6299 CNRS, Nantes, France; ^3^Université de Nantes, Nantes, France; ^4^Service de Gynécologie-Obstétrique, CHU de Poitiers, Poitiers, France; ^5^Institute for Transuranium Elements, European Commission Joint Research Centre, Karlsruhe, Germany; ^6^Service de Médecine Nucléaire, CHU de Nantes, Nantes, France; ^7^Service de Chirurgie Oncologique, Institut de Cancérologie de l’Ouest, Saint-Herblain, France; ^8^Service de Médecine Nucléaire, Institut de Cancérologie de l’Ouest, Saint-Herblain, France

**Keywords:** radioimmunotherapy, alpha-particles, HIPEC, ovarian peritoneal carcinomatosis, CD138

## Abstract

**Purpose:**

Ovarian peritoneal carcinomatosis is a pathology for which effective cures are currently lacking. New research protocols seek to eradicate residual micrometastases following cytoreductive surgery by using hyperthermic intraperitoneal chemotherapy (HIPEC) or radioimmunotherapy (RIT). This study aims to first develop alpha-RIT using an anti-CD138 mAb radiolabeled with an alpha-emitter, bismuth-213 (^213^Bi-B-B4) and HIPEC in a nude mouse model and second to compare and combine these techniques.

**Material and methods:**

A murine model of postoperative ovarian peritoneal carcinomatosis was established. A pilot group of six mice received an intraperitoneal injection of luciferase-tagged SHIN-3 cells and bioluminescence was measured every day. Cytoreductive surgery was performed at day 14 (*n* = 4) and 29 (*n* = 2). Because the residual bioluminescence signal measured after surgery was equivalent to that obtained 3 days after the graft, HIPEC or alpha-RIT treatments were applied 3 days after the graft. Ten mice were treated by HIPEC with cisplatine (37.5 mg/mL), 11 with 7.4 MBq of ^213^Bi-B-B4, seven with 11.1 MBq of ^213^Bi-B-B4, and 10 mice were treated with the combined therapy (HIPEC + 7.4 MBq of ^213^Bi-B-B4). Eleven mice received no treatment. Bioluminescence imaging and survival were assessed.

**Results:**

Alpha-RIT 7.4 MBq and 11.1 MBq significantly improved survival (*p* = 0.0303 and *p* = 0.0070, respectively), whereas HIPEC and HIPEC + alpha-RIT treatments did not significantly ameliorate survival as compared to the control group.

**Conclusion:**

Survival was significantly increased by alpha-RIT treatment in mice with peritoneal carcinomatosis of ovarian origin; however, HIPEC alone or in combination with alpha-RIT had no significant effect.

## Introduction

Epithelial ovarian carcinoma (EOC) is the leading cause of mortality linked to gynecological cancer in France with 3357 deaths in 2006 (1). The first line of treatment is based on a combination of maximal cytoreductive surgery and adjuvant chemotherapy with platinum salt and taxanes (2). Seventy-five percent of patients will experience cancer recurrence, and in the absence of complete remission in the initial period, the disease will become incurable (3). After initial treatment, the prognosis is closely correlated with residual tumor volume (4, 5). As EOC is mainly confined to the peritoneal cavity in the form of peritoneal carcinomatosis, several techniques including hyperthermic intraperitoneal chemotherapy (HIPEC) or radioimmunotherapy (RIT) are used to target the postoperative intraperitoneal residue.

HIPEC involves applying high concentrations of chemotherapy at cytotoxic temperatures to the intraperitoneal cavity during the perioperative cytoreductive period (6). A recent French phase III and IV multicenter retrospective cohort study of 566 patients treated with HIPEC found mortality and morbidity levels of 0.8 and 31.3%, respectively, for advanced and recurrent ovarian cancer (7). In addition, another multicenter retrospective study estimated that overall 4-year survival rates for patients treated with HIPEC was 75.6% compared to 19.4% for the control group (8), and recently a retrospective observational multi-institutional study was conducted showing that HIPEC results in encouraging survival rates for patients treated for a first relapse of ovarian cancer (9).

RIT is based on a radiopharmaceutical product composed of a specific tumor cell vector and a radioisotope. The radiopharmaceutical binds to an antigen which is overexpressed by the tumor cells and thus ensures selective irradiation. In this context, CD138 or Syndecan 1, a transmembrane receptor belonging to the heparan sulfate group overexpressed in ovarian tumors compared to healthy ovarian tissue, regardless of their status of chemoresistance or hormonal sensitivity (10, 11), could be a good target candidate. RIT is preferentially used on disseminated tumors or small-sized nodules as is the case with peritoneal carcinomatosis. A recent review by Tomblyn et al. summarized over 24 studies of RIT treatment of ovarian cancer in both preclinical and clinical studies (phases I–III) (12). The beta-particle emitters (iodine-131, rhenium-186, lutetium-177, and yttrium-90) were prioritized up until a phase III randomized study by Verheijen et al. in 2006 (13) which failed to establish an improvement in terms of survival only reduction of intraperitoneal relapses. One of the explanations lay in the choice of the radioelement (14). The development of alpha-particle emitters has enabled new studies, notably those using antibodies or antibody fragments (F(ab′)_2_) labeled with astatine-211, with the results showing good efficacy on tumor cell lysis with higher mean doses of radiotherapy absorbed (22 Gy) (15).

This study aims to target the residual pathology of ovarian peritoneal carcinomatosis by means of a monoclonal humanized anti-CD138 antibody radiolabeled with an alpha-particle emitter (bismuth-213) and to compare and combine this treatment with HIPEC.

## Materials and Methods

### Cell Line

The SHIN-3 cell line was established from a 56-year-old Japanese woman diagnosed with serous cystadenocarcinoma of the ovary and was provided by the Medical University of Nara, Japan (16). A cDNA sequence coding for Luciferase was obtained from the pGL3 vector (Promega). The fragment was subcloned into the retroviral vector pMX (17) and retroviral transduction of SHIN-3 cells was performed by standard molecular methods. SHIN-3-Luc+ cells were grown in RPMI 1640 medium (Sigma-Aldrich) supplemented with 10% heat-inactivated fetal calf serum (PAA), 2 mM glutamine (Invitrogen), 100 U/mL penicillin (Invitrogen), and 100 μg/mL streptomycin (Invitrogen) at 37°C, 5% CO_2_, and 100% humidity. The doubling time of SHIN-3-Luc+ cells was 29 h.

### Cellular Expression of CD138 and CEA by Flow Cytometry

To measure cellular expression of CD138 and CEA on SHIN-3-Luc+ cells, 200,000 cells were incubated for 1 h at 4°C with anti-CD138 (B-B4, Diaclone), anti-CEA (T84.66) antibodies or with isotype control mouse IgG1 (BD Biosciences) at 10 μg/mL, followed by a secondary phycoerythrin conjugated anti-rat antibody (Jackson Immuno Research) for 1 h. Analysis was carried out using a FACSArray flow cytometer (Becton Dickinson). Data analysis was conducted using Flowjo software.

### Ovarian Peritoneal Carcinomatosis Mouse Model

NMRI-nu (nu/nu) mice were purchased from Janvier and housed under conventional conditions at the UTE animal facility (SFR François Bonamy, IRS-UN, University of Nantes, license number: B-44-278). Experiments were approved by the local veterinary committee (License number: CEEA.2013.2).

Mice were 7–10 weeks old at the time of experiments and were grafted using intraperitoneal injection with 5 × 10^6^ SHIN-3-Luc+ cells in 0.3 mL of phosphate buffer saline.

### Monitoring of Ovarian Peritoneal Carcinomatosis by Bioluminescence

One hundred microliters of luciferin solution (12 mg/mL) was injected intraperitoneally 5 min before the acquisition of images (17). The mice were then anesthetized in an airtight container containing 3.5% of isoflurane (Abbott Laboratories) and isoflurane was maintained using a nose cone delivery system when passing under the “photon Imager” camera (Biospace Lab). Images were taken for each mouse for 1 min on both sides (in prone and supine positions). All images were analyzed (software M3viewer 2.2, Biospace Lab) while determining a specific region of interest (ROI) encompassing the entire surface of the mouse (except tail). For each mouse, each time point was the sum of the photon counts obtained in both prone and supine positions.

### Cytoreductive Surgery and Postoperative Residual Volume Determination

**Figure 1:**
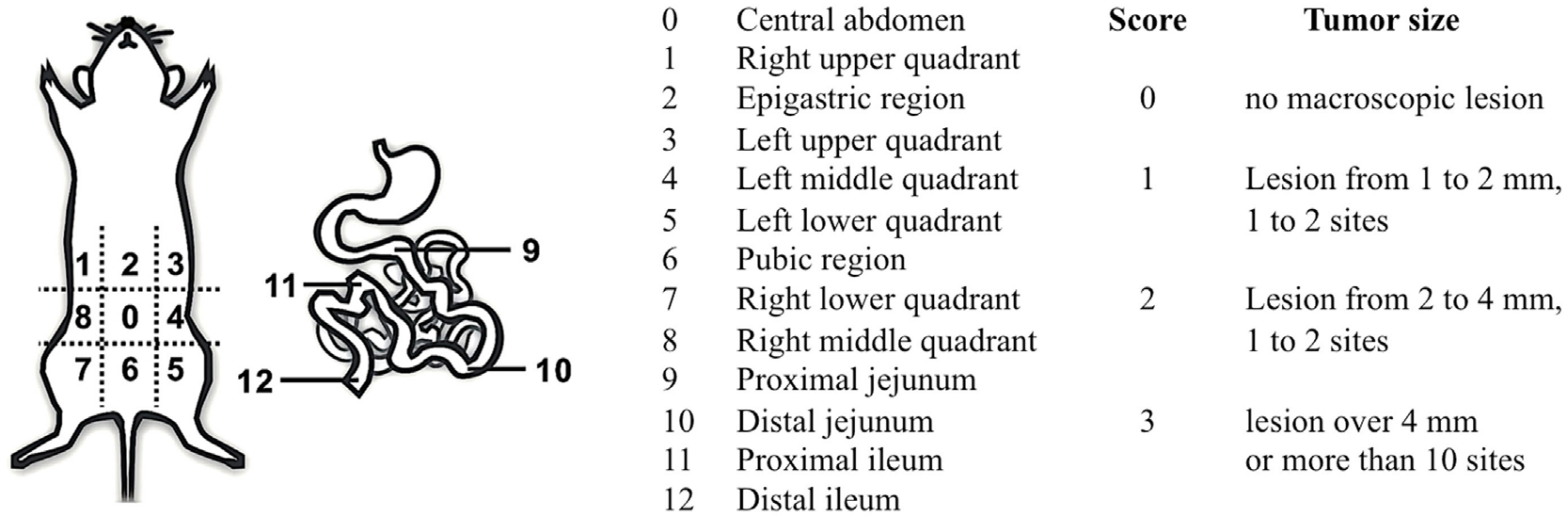
**Peritoneal carcinomatosis index (PCI) in mice**. Exploration of the 13 standard regions of the abdominal cavity was carried out using magnifiers according to the descriptions of tumor damage by PCI scoring adapted from those of Klaver et al. (18) and Sugarbaker (6).

Cytoreductive surgery was performed 14 days (*n* = 4) and 29 days (*n* = 2) after injection of 5 × 10^6^ SHIN-3-Luc+ cells. Mice were anesthetized following intraperitoneal injection of 100 μL/10 g anesthetic solution (consisting of 1 mL ketamine at 100 mg/mL, Panpharma; 0.5 mL xylazine at 20 mg/mL, Bayer; and 8.5 mL PBS). A midline laparotomy was performed. Thorough exploration of the abdominal cavity was carried out using magnifiers for the 13 standard regions defined by Sugarbaker (6) and adapted to rodents by Klaver et al. (18). For the present study, descriptions of tumor damage by Peritoneal Carcinomatosis Index (PCI) scores are summarized in Figure 1. Scores from 0 to 3 were defined for each region (0): no macroscopic tumor; (1): lesion from 1 to 2 mm, 1 to 2 sites; (2): lesion from 2 to 4 mm, 1 to 2 sites; and (3): lesion over 4 mm or more than 10 sites. The total PCI score was calculated as the sum of the score for each region and could thus range from 0 to 39. All observed nodules were resected using an electric scalpel blade. At the end of the procedure, the quality of the exeresis was described and classified using a “completeness of cytoreduction” (CC) score adapted from Sugarbaker (6) and Klaver et al. (18): CC0: absence of macroscopic residue, CC1: tumoral residue below 2.5 mm, and CC2: tumoral residue above 2.5 mm. The lining was sutured along two planes. Postoperative analgesia was provided by i.p. injection of 0.20 μL buprenorphine at 0.3 mg/mL (Axience), repeated after 12 h, followed by oral administration of anti-inflammatory drugs (Ibuprofen, 7.5 mg/kg, Wyeth).

Bioluminescent imaging was carried out after cytoreductive surgery while the animals were still under anesthesia to evaluate the correlation between postoperative residual tumor volume and bioluminescence signal.

### Determination of the Optimal Time of Treatment After Engraftment

Eleven mice were grafted with 5 × 10^6^ SHIN-3-Luc+ cells (control mice). Bioluminescence imaging was performed 1, 2, 3, 7, 11, 14, 18, and 21 days after engraftment. To determine the time after engraftment mimicking the postoperative residual tumor volume, i.e., time of HIPEC/alpha-RIT treatment, bioluminescence signals were compared to those obtained from the four and two mice which had cytoreductive surgery 14 and 29 days after engraftment, respectively.

### HIPEC

Mice were anesthetized in an induction chamber and maintained using a mask (isoflurane 1.5%). Mice were placed on a heat pad and a 1–2-cm midline incision was made. The instillation tube and a multiperforated suction appliance were inserted inside the abdomen and cisplatin was administered (Generic Merck, 37.5 mg/mL or 75 mg/m^2^). The Erlenmeyer flask containing the cisplatin was placed in a water bath heated to 49°C, and administration was achieved using a peristaltic pump (3 mL/min). The temperature of the intraperitoneal solution was 39°C as previously described by Muller et al. (19) and was regularly checked using a thermal probe. The tubes were often repositioned in the abdominal cavity and the abdomen was regularly massaged. After 60 min, the lining was sutured along two planes using Vicryl^®^ 4-0. The animal recovered consciousness under a heat lamp and postoperative analgesia was provided as previously described.

### B-B4 Radiolabeling with Bismuth-213 and *In vivo* Alpha-RIT

B-B4 was conjugated with the chelating agent CHX-A"-DTPA (Macrocyclics) according to the protocol described by Supiot et al. (20). The bismuth-213 was eluted from the actinium-225/bismuth-213 generator using a solution composed of 500 μL of 0.2 M NaI and 500 μL of 0.2 N HCl (21). One hundred micrograms of antibody, along with 195 μL of 4 M NaOAc buffer solution and 75 μL of 20% ascorbic acid were added. After 10 min at 37°C, the antibody was purified of free bismuth-213 on a PD-10 desalting column (GE Healthcare) in 0.3 mL PBS fractions. The fractions with highest activity were combined and the total activity was assessed using a NaI detector (X-ray test). Next, ^213^Bi-B-B4 was filtered through a 0.2 μm filter (Whatman, Anotop 10) before being injected i.p. (from 350 to 430 μL). The specific activity after filtration was 0.48 ± 0.07 MBq/μg of mAb.

Three days after engraftment, for alpha-RIT, 7.4 MBq or 11.1 MBq of ^213^Bi-B–B4 were injected and for the HIPEC + alpha-RIT group, 7.4 MBq of ^213^Bi-B–B4 were injected immediately after HIPEC surgery, either during or after recovery.

### Animal Monitoring

Every week, the mice were weighed, clinically assessed and bioluminescence imaging was carried out. The animals were sacrificed if they met the ethical conditions of euthanasia; signs of abnormal behavior (difficulty moving and feeding), excessive weight loss, major ascites. Next, an autopsy was performed on all mice. For each animal the tumor volume was assessed and assigned a PCI score. The endpoint of the study was set at 90 days. Blood samples were collected into tubes containing 5% EDTA. Platelets, white, and red blood cells were counted by a quantitative automated hematology analyzer (Melet-Schloesing).

### Statistical Analysis

Statistical analysis was conducted via GraphPad Prism software (GraphPad Software Inc., San Diego CA). Analysis of the different groups of treated mice at the end of the study was conducted using a two-way repeated measures variance analysis (two-way ANOVA). Corrections for the multiple groups were carried out using Bonferoni tests. Comparison of postoperative average signals of bioluminescence was obtained using a Student’s *t*-test with 4 degrees of freedom. Finally, survival was examined by means of a Kaplan–Meier survival curve and log-rank test analysis.

## Results

### Expression of CD138 and CEA by SHIN-3-Luc+ Cells

**Figure 2:**
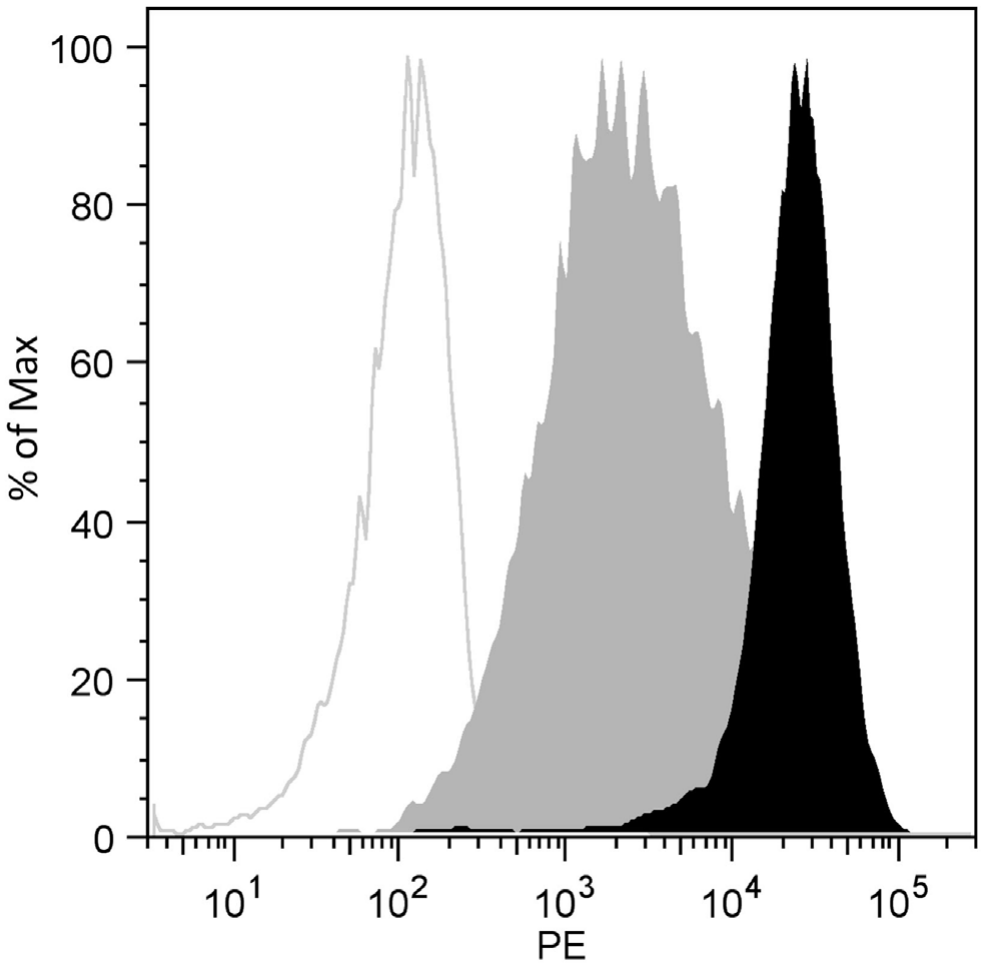
**CD138 and CEA expression by flow cytometry**. The SHIN-3-Luc+ cells were incubated in the presence of a saturating dose of anti-CD138 (IgG1 mouse, B-B4, black), anti-CEA (IgG1 mouse, T84.66; gray), or an isotype control antibody (IgG1 mouse, gray line). One representative experiment (out of 3 independent experiments) is shown.

The expression of CD138 by SHIN-3-Luc+ cells was compared to that of CEA which has previously been used as a RIT target (22). The data relating to flow cytometry analysis of SHIN-3-Luc+ cells (Figure 2) show that CD138 expression was 10 times higher than that of CEA (MFI of 26,825 and 2,530, respectively).

### Ovarian Peritoneal Carcinomatosis Model

**Figure 3:**
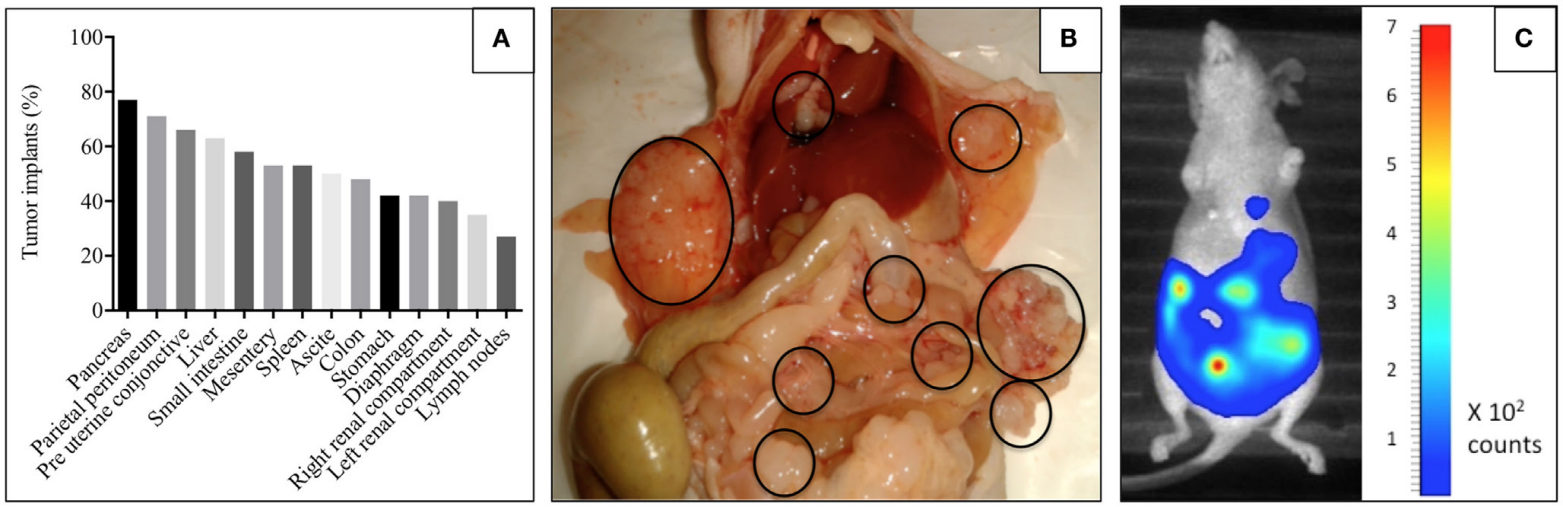
**SHIN-3-Luc+ ovarian peritoneal carcinomatosis model in nude mice**. **(A)** Shows the frequency in the localization of intra-abdominal tumoral damage in nude mice after grafting with 5 × 10^6^ SHIN-3-Luc+ cells. These evaluations were carried out on all mice during cytoreductive surgery or following death. **(B)** Is a photograph illustrating the distribution of tumoral lesions in the abdominal cavity in a nude mouse with a graft of 5 × 10^6^ SHIN-3-Luc+ cells after sacrifice. Circles highlight visible tumor lesions. **(C)** Is a photograph illustrating the bioluminescence signal measured with the photon Imager.

Female nude mice were injected with 5 × 10^6^ SHIN-3-Luc+ cells. The first ascitic fluid was apparent on day 27 after grafting. The distribution of nodules was very heterogeneous and disseminated as shown in Figures 3A,B, closely mimicking human ovarian peritoneal carcinomatosis. A similar heterogeneity and dissemination were observed from the bioluminescence signal acquisition (Figure 3C).

### Determining the Correspondence Between Postoperative Residual Tumor Volume and Bioluminescence Signal

Currently, therapies such as HIPEC or RIT are considered as adjuvant therapies applied immediately before or after cytoreductive surgery. In order to treat animals in a comparable manner while avoiding cytoreductive surgery, the postoperative residual tumor volume was assessed by bioluminescence imaging on 6 mice grafted with 5 × 10^6^ SHIN-3-Luc+ cells. Four mice with a total PCI score ranging from 2 to 5 (2, 3, 3, and 5) had cytoreductive surgery on day 14. After the procedure, the CC was between 0 and 1 (0, 0, 1, and 1). Two other mice with a total PCI score of 6 and 11, respectively, had cytoreductive surgery on day 29. Both mice had a CC score of 1 after the surgery.

Bioluminescence signals obtained postoperatively from these mice operated 14 and 29 days after engraftment were, respectively, 4.9 × 10^5^ ± 3.3 × 10^5^ counts (*n* = 4) and 3.7 × 10^5^ ± 1.7 × 10^5^ counts (*n* = 2). The comparison of these two means using a Student’s *t*-test with 4 degrees of freedom did not show any significant difference (*p* < 0.005). Hence, a postoperative bioluminescence signal was established at 4.3 × 10^5^ counts accumulated per mouse corresponding to postoperative residual tumor volume (i.e., CC between 0 and 1).

### Determining by Bioluminescence the Day After Engraftment Mimicking Postoperative Residual Tumor Volume

**Figure 4:**
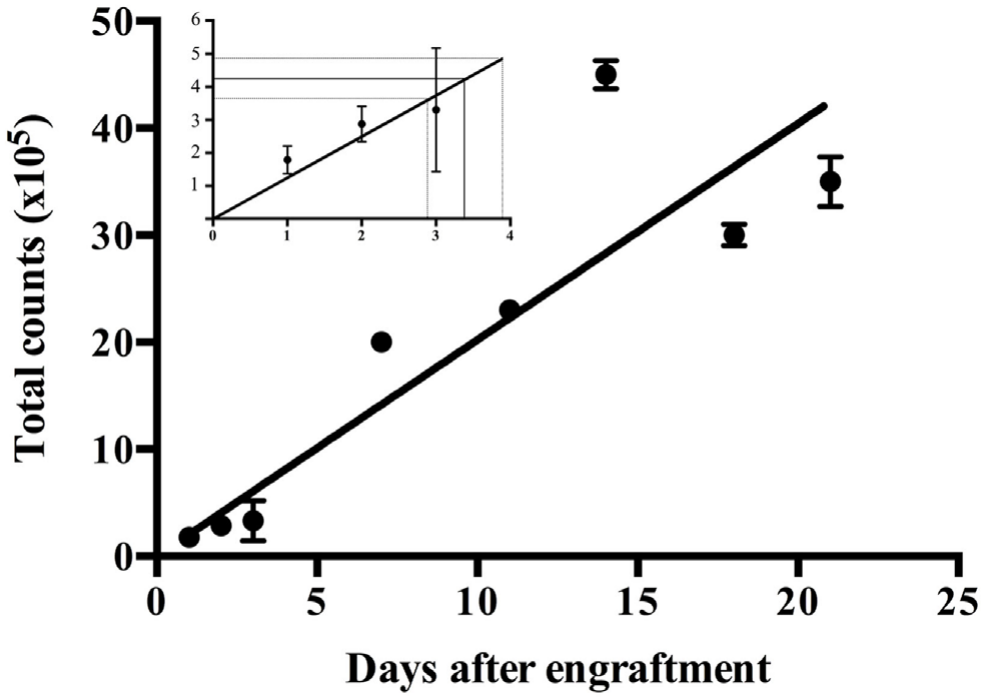
**Progression of the bioluminescence signal according to the day after engraftment**. Bioluminescence signals were measured for 11 mice up until 21 days after engraftment. The insert focuses on the first 4 days after engraftment. The day after engraftment mimicking the postoperative residual tumor volume, i.e., the disease status after cytoreductive surgery, was determined by reporting the postoperative bioluminescence signals on the *y*-axis to against the corresponding day on the *x*-axis.

Bioluminescence was measured for 11 mice from 1 to 21 days after engraftment. Figure 4 shows the progression of the disease expressed as the evolution of the bioluminescence signals according to the day after the engraftment. Postoperative bioluminescence signals of 4.9 × 10^5^ counts, 3.7 × 10^5^ counts and 4.3 × 10^5^ counts reported on the *y*-axis of Figure 4 (see insert), correspond on the *x*-axis to an interval of one day, just before day 3 and day 4, after engraftment. Day 3 was defined as the day after engraftment mimicking the postoperative residual tumor volume, i.e., the disease status after cytoreductive surgery.

To verify the tumor status 3 days after engraftment, five mice grafted with 5 × 10^6^ SHIN-3-Luc+ cells were sacrificed after bioluminescence imaging 3 days later (3.9 × 10^5^ ± 1.6 × 10^5^ counts). As shown in Table 1, the total PCI score for each mouse was 3, 3, 1, 2, and 1. All tumor lesions were less than 2 mm as the PCI score for each region was never >1. Thus, the tumor status of each mouse at day 3 was equivalent to a CC score <1.

**Table 1:** **PCI scoring 3 days after engraftment in the 13 standard regions (*n* = 5)**.

	Region number													
	0	1	2	3	4	5	6	7	8	9	10	11	12	Total PCI
Mouse 1	0	0	0	1	0	0	1	0	1	0	0	0	0	3
Mouse 2	0	0	0	0	0	0	0	1	1	0	0	0	1	3
Mouse 3	0	0	1	0	0	0	0	0	0	0	0	0	0	1
Mouse 4	0	0	0	1	0	0	0	1	0	0	0	0	0	2
Mouse 5	0	0	0	1	0	0	0	0	0	0	0	0	0	1

### Treatment of Residual Tumor Volume

In two independent experiments, 49 mice were randomly assigned to the different treatment groups which were initiated on day 3 after the graft. A first experiment included control (*n* = 6), HIPEC only (*n* = 5), alpha-RIT 7.4 MBq (*n* = 6), and HIPEC + alpha-RIT 7.4 MBq (*n* = 5) groups. The second experiment included control (*n* = 5), HIPEC only (*n* = 5), alpha-RIT 7.4 MBq (*n* = 5), alpha-RIT 11.1 MBq (*n* = 7), and HIPEC + alpha-RIT 7.4 MBq (*n* = 5) groups. During HIPEC, the intraperitoneal temperatures of both the HIPEC and the HIPEC + alpha-RIT groups remained at a stable average temperature of 39°C. Two intraoperative deaths were observed.

### Clinical Results

**Figure 5:**
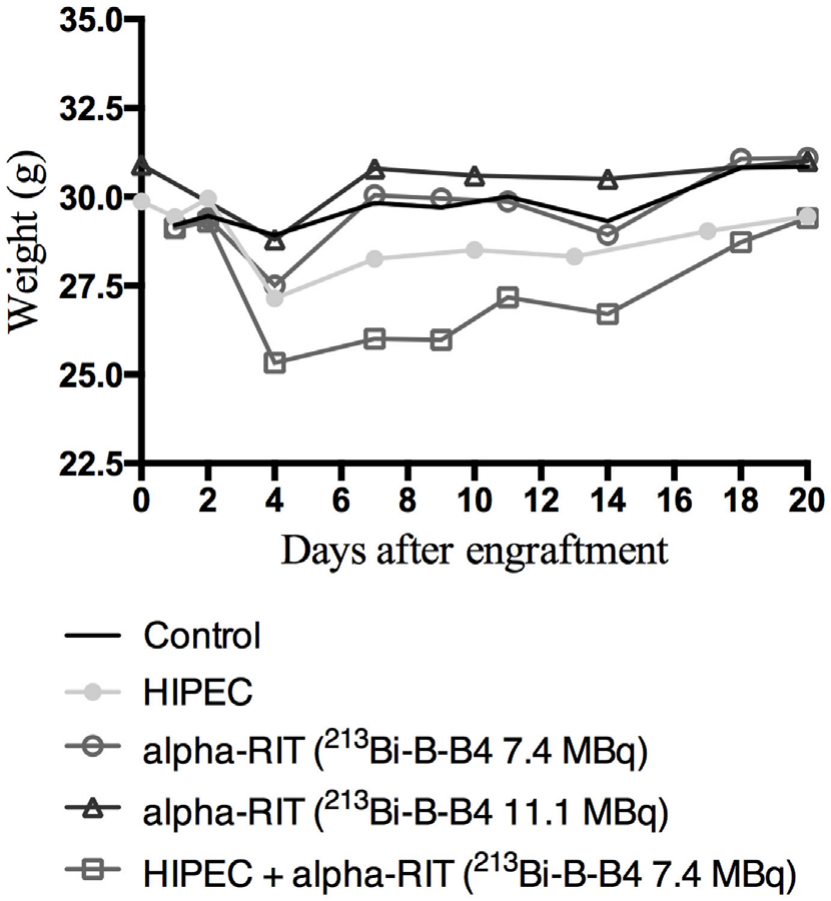
**Weight monitoring of mice after treatment**. This figure illustrates the weight changes for each group (control *n* = 6, HIPEC *n* = 5, alpha-RIT 7.4 MBq *n* = 6, alpha-RIT 11.1 MBq *n* = 7, and HIPEC + alpha-RIT 7.4 MBq *n* = 5) according to the day after engraftment.

The mice receiving HIPEC treatment alone had a weight loss of 2 g at day 4 after the treatment, equal to that of the alpha-RIT groups, while those from the HIPEC + alpha-RIT group experienced a weight loss of 4 g (Figure 5). At day 7, the mice treated with alpha-RIT recovered their initial weights. On the contrary, the weights of HIPEC and HIPEC + alpha-RIT mice were never restored (Figure 5).

### Bioluminescence Results and PCI-Scoring

**Figure 6:**
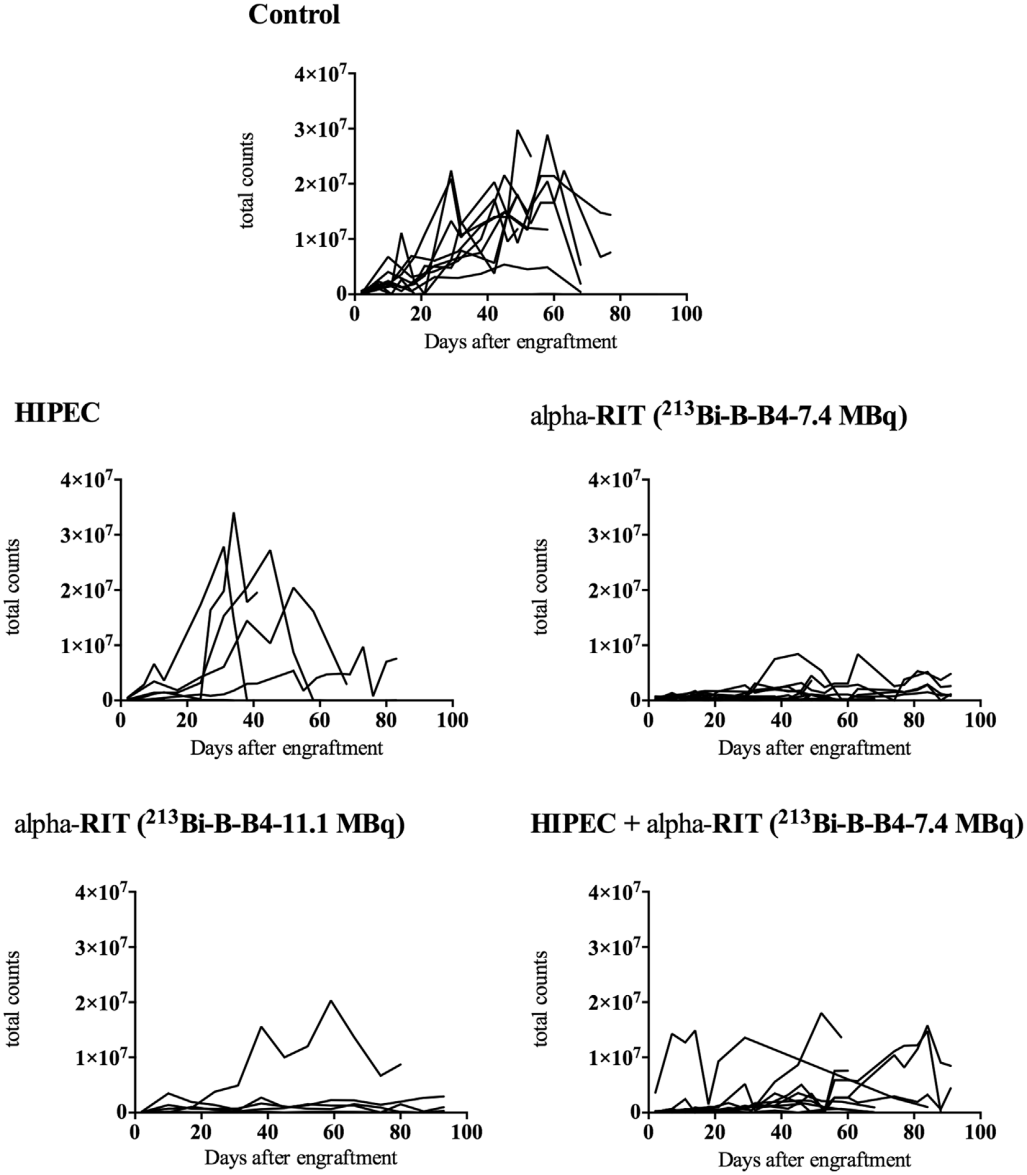
**Changes in bioluminescence signal in relation to treatment received**. This figure shows changes in bioluminescence signal for each group treated (control *n* = 11, HIPEC *n* = 7, alpha-RIT 7.4 MBq *n* = 11, alpha-RIT 11.1 MBq *n* = 7, and HIPEC + alpha-RIT 7.4 MBq *n* = 10) as a function of time (days after engraftment). For each mouse and each time point, the sum of the bioluminescence signal obtained in both the prone and the supine positions is represented.

Figure 6 shows changes in the bioluminescence signal for each mouse in relation to the treatment received. These results show that the bioluminescence signal was much weaker for the groups having received alpha-RIT. Only the curves relating to HIPEC come close to matching those of the mice receiving no treatment.

At sacrifice, the abdominal cavity of the mice was explored to establish the PCI score. The average PCI score was compared to the average maximal bioluminescence signal, with results showing a correlation of the two parameters. Indeed, for the control group an average PCI of 32.4 was found for a maximal average bioluminescence signal of 2.7 × 10^7^ photon counts. For the HIPEC group only a PCI average of 26.6 with an average maximal bioluminescence signal of 2.2 × 10^7^ counts, for the combined HIPEC + alpha-RIT group a PCI average of 28.5 for an average maximal bioluminescence signal of 1.2 × 10^7^ counts, and for the alpha-RIT group at 7.4 MBq an average PCI of 10.6 with a maximal average bioluminescence signal of 0.45 × 10^7^counts.

### Toxicity

**Figure 7:**
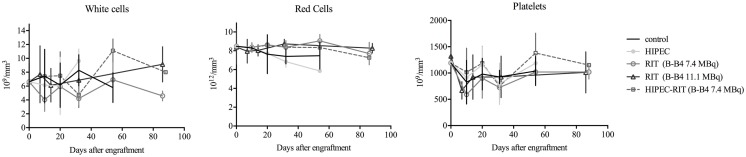
**Measurement of blood cell counts according to treatment groups**. This figure shows the average changes in red blood cell levels, leukocytes, and platelets as a function of time (days after engraftment) and treatment group.

In terms of blood toxicity, Figure 7 shows a transient but nonetheless insignificant reduction in platelets and white blood cells for all treatments. For HIPEC alone, a substantial reduction in red blood cells was highlighted 51 days after treatment with no hemorrhage being observed.

### Survival

**Figure 8:**
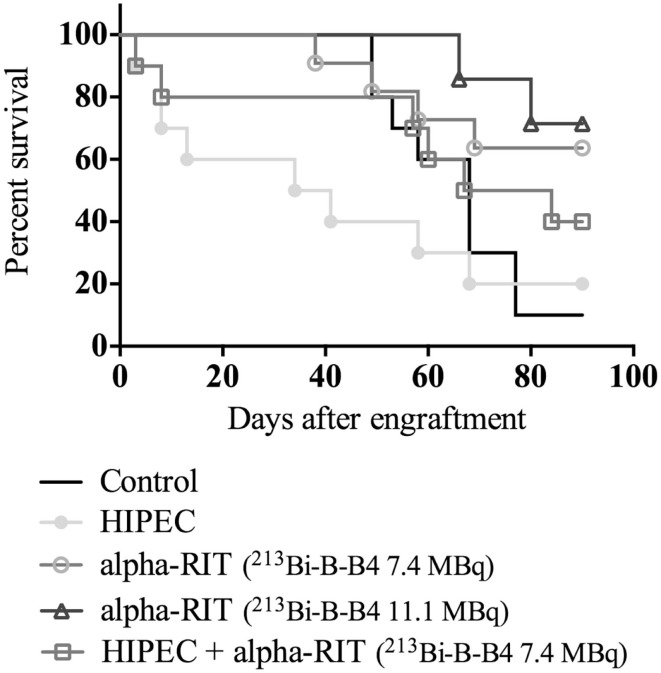
**Survival rates according to treatment groups**. This figure shows the Kaplan–Meier survival curves of mice as a function of treatment by day 3, and according to the number of days.

The survival curves are shown in Figure 8. The first deaths occurred intraoperatively for the mice from the HIPEC + alpha-RIT group and the HIPEC group (two deaths on the day of the procedure). Similarly, the two groups receiving HIPEC experienced the most premature deaths (two on day 8 after the graft and one on day 13 after the graft), without evident causes being established via autopsies. Median survival was 68 days for the control group, 75.5 days for the combined HIPEC + alpha-RIT group and 37.5 days for the HIPEC group. Median survival rates for the groups having received RIT alone cannot be calculated, given that over 50% of the mice were still alive at the end of the experiment. Indeed, at 90 days, seven mice out of 11 (63.6%) were still alive in the group receiving alpha-RIT 7.4 MBq and five out of seven (71.4%) in the group with alpha-RIT 11.1 MBq. In this last group, one mouse was sacrificed due to major weight loss and the necropsy could only establish two small millimeter-sized tumoral nodules. As compared to the control group curve, HIPEC and HIPEC + alpha-RIT curves were not significantly different whereas alpha-RIT 7.4 MBq and alpha-RIT 11.1 MBq were significantly different (*p* = 0.0303 and *p* = 0.0070, respectively).

## Discussion

The present preclinical study is the first to compare and combine HIPEC and alpha-RIT for the treatment of ovarian peritoneal carcinomatosis in a model mimicking postoperative residual tumor illness. Alpha-RIT, combining anti-CD138, and bismuth-213 showed a substantial increase in overall survival in correlation with low bioluminescence signals. However, HIPEC did not show significant improvements in terms of survival but did show a reduction in median survival and disease progression measured by bioluminescence, similar to the control group. Finally, a combination of both therapies did not show significant improvements in overall survival. With an uncertain prognosis (3) and based on residual tumor volume after surgery (4, 5), the development of innovative therapies in EOC is required for reliable and reproducible preclinical models. Our model based on SHIN-3 cells offers the advantage of rapidly establishing peritoneal carcinoma disease (Figure 3) that is identical to that found in advanced ovarian cancer.

Cytoreductive surgery plays a key role in the treatment of EOC, and disease prognosis depends on the degree of its success (5). The possibility of applying this surgery to preclinical models on animals has been demonstrated, notably with rats for colon cancer (18, 23) but also with mice (24). Nevertheless, the teams studying therapies targeting postoperative residual tumor volume seek to amend surgery, and administer their therapies at an early stage of the graft, often day 1 or day 2, under the assumption that the tumor volume must be the same at day 1 and day 2 (24), without taking into account the viability of the graft at this early stage. Our cytoreductive surgery model performed on 6 mice has enabled us to determine the correspondence between the bioluminescence signal obtained immediately after surgery and the one obtained after engraftment. We therefore decided to treat the animals on day 3 in order to be as close as possible to the postoperative state of the tumor. Finally, cytoreductive surgery was performed in the context of PCI scoring between 2 and 11, corresponding to advanced phases of disease, with the results of cytoreduction (CC 0–1) similar to those obtained in the clinic (7).

RIT has been used for EOC treatment since the 1980s (25) with constant clinical inefficiency of RIT using beta particles (13). However, the efficacy of alpha particle vectorization has been demonstrated in preclinical ovarian cancer models (15), subsequently leading to a phase I clinical trial (26). In our study, we used bismuth-213 as an alpha-particle emitter. Although the half-life of bismuth-213 (45 min) seems theoretically more advantageous than that of astatine-211 (7.2 h) with the aim of restricting intraperitoneal cavity activity, a study by Gustafsson has shown similar effects between two alpha particle emitters (27). The activities of bismuth-213 used in our study (7.4 and 11.1 MBq) did not result in acute blood toxicity and are consistent with a study by Song et al. (28) which showed, for an antibody injected by i.p., a MTD of 35 MBq for a nude mouse of 30g. In the same manner, HIPEC, developed to improve the benefits of intraperitoneal chemotherapy while amending inflammatory procedures, may be worthwhile as a direct application of intraoperative RIT on difficult or partial areas of resection so as to target residual disease.

CD138 or syndecan-1 belongs to the heparan sulfate proteoglycan family and is often targeted during RIT treatments. Two studies have examined the expression of CD138 in ovarian cancer: the first (10) revealed the presence of CD138 in benign tumors and the borderline of the ovary, and in adenocarcinomas (present in epithelial and stromal cells), while not being expressed by healthy ovarian tissue. The second study showed higher CD138 expression in all cancer types compared to healthy ovarian tissue, without any correlation in terms of chemoresistance or exposure to estrogens (11). Therefore, we considered CD138 as a potential innovative target antigen for the treatment of ovarian cancer by RIT, in the same way as other emerging targets such as Her2 (29) or VEGF (30). This choice was strengthened by the results that we obtained in this study on CD138 expression and RIT. CD138-targeted alpha-RIT results in a marked difference in terms of bioluminescence signals (Figure 6). Furthermore, as shown in Figure 8, we observed a significant increase in survival (63.6 and 71.4%, respectively) at 90 days for the alpha-RIT 7.4 MBq (*p* = 0.0303) and alpha-RIT 11.1 MBq (*p* = 0.0070) groups compared with 10% for the untreated mice. In addition, the mice treated with alpha-RIT experienced lower weight loss than the other groups, and a markedly lower tumor volume at necropsy (PCI average of 10.6).

The HIPEC technique used in this study was designed to reproduce as faithfully as possible the technique used on humans with EOC, consisting of a semiopen procedure with cisplatin perfusion at 3 mL/min (24). In France, cisplatin is the most frequent chemotherapeutic product used in ovarian HIPEC (7). Doses >80 mg/m^2^ result in renal failure (31). In this study, the concentration of cisplatin was 75 mg/m^2^ or 2 mg/kg. The upper intraperitoneal temperature limit tolerated was 40°C, as bowel lesions can occur in rodents at higher temperatures (32). In a preliminary study, we demonstrated that using temperatures above 40°C on SHIN-3 cells required an increase in the concentration of cisplatin in order to obtain a similar cytotoxic effect (19).

In the present study, the group receiving HIPEC alone did not show any differences in terms of bioluminescence signal (Figure 6) or overall survival (Figure 8) compared to the control group. Median survival of these animals is even lower than that of the control group: 34 versus 68 days. There is a paucity of mouse studies evaluating cisplatin-based HIPEC in EOC. A study on the impact of the addition of sodium arsenite and variations in temperature in cisplatin HIPEC did not include survival as an assessment criterion and all the animals were euthanized from day 1 (24). In preclinical studies on colon cancer, HIPEC is associated with intense toxicity and a general worsening state, including lethargy, substantial weight loss, and bacterial translocations (33). However, we did not observe intense toxicity in our group of animals treated with HIPEC. Indeed, on average, initial weight loss of 2 g was observed, which was equivalent to the alpha-RIT-treated group, and below that of the combined HIPEC + alpha-RIT-treated group. Similarly, blood sample analysis did not indicate greater toxicity in this group. In 2010, Klaver et al. compared the survival of cancer bearing rats treated by cytoreductive surgery alone or combined cytoreductive surgery and HIPEC, with greater survival for animals treated by HIPEC surgery (18). Next we combined HIPEC and alpha-RIT for the first time in an animal model. The alpha-RIT injection was carried out immediately after HIPEC, and only one mouse had to be reinjected with alpha-RIT due to leakage of the radiolabeled vector. While the bioluminescence curve appeared different to the control group (Figure 6), no significant difference was noted in terms of survival (Figure 8). Examination of the survival curve of the HIPEC + alpha-RIT group, showed early deaths potentially due to the toxicity of the combined treatments. This hypothesis is supported by the higher initial weight loss in this group (4 g), but did not correlate with toxicity as measured by blood sample analysis (Figure 7). Second, this group displayed fewer deaths and mice appeared similar to those of the groups treated with alpha-RIT. Currently, no study has associated these two treatments. Only one Dutch team has compared them in two separate studies. The first used rats with peritoneal carcinoma of colorectal origin treated by HIPEC or alpha-RIT after cytoreductive surgery was focused on animal survival (34). A second study, using the same model, focused on the impact of these treatments on abdominal healing after surgery (35). In the first study, concerning survival (34), the results are in favor of alpha-RIT, with a non-significant increase in survival for the HIPEC group compared to the control group.

## Conclusion

The present study indicates the potential therapeutic effect of alpha-RIT in EOC. It demonstrates that the use of postoperative alpha-RIT in a context mimicking the residual tumor after cytoreductive surgery dramatically increased survival rates of mice with peritoneal carcinomatosis of ovarian origin. Under the same conditions, HIPEC treatment alone or combined HIPEC + alpha-RIT did not offer significant improvements to survival rates. We believe alpha-RIT provides a potentially interesting alternative adjuvant treatment along with cytoreductive surgery in the treatment of ovarian cancer. Furthermore, our study has extended the scope of available target antigens for RIT, showing that CD138 can be used effectively to target peritoneal carcinoma cells.

## Author Contributions

Conceived and designed the experiments: AD, SG, and MC. Performed the experiments: AD, SG, CM, J-MC, and MC. Analyzed the data: AD, SG, J-MC, and MC. Wrote the paper: AD, SG, M-HG, and MC.

## Conflict of Interest Statement

The authors declare that the research was conducted in the absence of any commercial or financial relationships that could be construed as a potential conflict of interest.
